# TGF-β1 Induced Transdifferentiation of RPE Cells is Mediated by TAK1

**DOI:** 10.1371/journal.pone.0122229

**Published:** 2015-04-07

**Authors:** Zeev Dvashi, Mordechai Goldberg, Orit Adir, Michal Shapira, Ayala Pollack

**Affiliations:** Kaplan Medical Center, Rehovot, affiliated with Hadassah-Hebrew University of Jerusalem, Rehovot, Israel; National Eye Institute, UNITED STATES

## Abstract

**Background and Aim:**

Proliferative vitreoretinopathy (PVR) is an active process that develops as a complication upon retinal detachment (RD), accompanied by formation of fibrotic tissue. The main cells involved in the development of fibrotic tissue during PVR are the retinal pigment epithelial (RPE) cells. The RPE cells undergo epithelial-mesenchymal transition (EMT) which leads to complex retinal detachment and loss of vision. Transforming growth factor-β1 (TGF-β1) is considered as the main player in the EMT of RPE cells, even though the mechanism is not fully understood. This study was performed to determine the possible involvement of transforming growth factor β activated kinase 1 (TAK1) in the EMT process of the RPE cells.

**Methodology:**

ARPE-19 Cells were treated with 5Z-7 oxozeaenol (TAK1 inhibitor) or SB431542 (TGF-β1 receptor kinase inhibitor) followed by TGF-β1 stimulation. Immunofluorescence, scratch assay Real time PCR and collagen contraction assay assessed the EMT features. The phosphorylation of Smad2/3 and p38 was examined using western blots analysis.

**Results:**

This study demonstrates that stimulation of RPE cells with TGF-β1 increases α-SMA expression, cell migration and cell contractility, all of which are EMT features. Remarkably, addition of TAK1 inhibitor abolishes all these processes. Furthermore, we show hereby that TAK1 regulates not only the activation of the non-canonical cascade of TGF-β1 (p38), but also the canonical cascade, the Smad2/3 activation. Thus, the outcome of the TGF-β response in RPE cells is TAK1 dependent.

**Conclusions/Significance:**

This work demonstrated TAK1, a component of the non-canonical pathway of TGF-β1, is a key player in the EMT process, thus provides deep insight into the pathogenesis of PVR. The ability to halt the process of EMT in RPE cells may reduce the severity of the fibrotic response that occurs upon PVR, leading to a better prognosis and increase the probability of success in RD treatment.

## Introduction

Proliferative vitreoretinopathy (PVR) is an active process that develops as a complication during retinal detachment (RD) and it is the most common cause of surgical failure upon RD treatment [1]. PVR is a dynamic process characterized by the formation of fibrotic tissue on the detached retina, preventing the reattachment of the retina and finally may cause blindness [2]. Retinal pigment epithelial (RPE) cells, which are normally located in the external cell layer of the retina, are the most critical contributors to the development of fibrotic diseases of the eye. During PVR, RPE cells undergo transformation into fibroblast-like cells through a process known as the epithelial-mesenchymal transition (EMT) [3]. In the process of converting from epithelial into mesenchymal cells, they lose their epithelial characteristics such as specialized cell-to-cell contact, and acquire migratory mesenchymal properties [4]. These processes are mediated by the expression of cell surface molecules, cytoskeletal reorganization, and extracellular matrix (ECM) components [5],[6]. EMT can be triggered by different signaling molecules such as epidermal growth factor (EGF) and fibroblast growth factor (FGF), however transforming growth factor β-1 (TGF-β1) is considered the main regulator of EMT [7–9].

TGF-β-mediated EMT has been observed in a variety of cell types, including lens epithelial cells, corneal epithelial cells and others [10]. TGF-β is a multifunctional cytokine with an array of biological effects such as cell growth, differentiation, immunomodulation by two-edged sword effect, oxidative stress and Endoplasmic Reticulum (ER) stress[11, 12]. Intracellular signaling downstream to the TGF-β receptor complexes is mediated by the Smads family, the canonical pathway [13]. Recent reports have demonstrated that transforming growth factor β activated kinase 1 (TAK1), a member of the mitogen-activating protein (MAP) kinase kinase kinase family, is involved in the TGF-β signaling in the non-canonical pathway [14–16]. TAK1 is a serine/threonine kinase that is rapidly activated by TGF-β1 and subsequently activates other MAP kinases such as p38 [17, 18]. Moreover, studies indicate that TAK1 can regulate TGF-β-induced activation of Smad signaling by inducing Smad7 expression and also interfering with R-Smad transactivation by direct interaction with the MH2 domain of Smad proteins[19]. In addition to the role of TAK1 in the regulation of Smad function, there is cross-talk between the Smad and downstream targets of TAK1 such as p38 MAPK and ATF2 in the regulation of certain TGF-β1 target genes expression [13, 14]. Even though TAK1 activation is associated with TGF-β1 signaling, it is well known that its activation can also be caused by various stimuli including: environmental stress, pro-inflammatory cytokines such as tumor necrosis factor-alpha (TNF-α), interleukin (IL)-1 and lipopolysaccharides (LPS)[20]. Activated TAK1 can transduce signals to several downstream signaling cascades, including the MKK4/7-JNK, MKK3/6-p38 MAPK, and Nuclear Factor-kappa B (NF-kB)-inducing kinase (NIK)-IkB kinase (IKK) [21].

In this study we examined the role of TAK1 during EMT of RPE cells and the fibrotic response which maybe applicable to PVR. We demonstrate hereby that TAK1 acts as a critical player in the regulation of RPE cells during EMT. Applying TGF-β1 on human ARPE-19 cells in culture and utilizing various experimental approaches we show that inhibition of TAK1 reduces cell migration, α-SMA expression and cell motility, all of which are considered hallmarks of fibrosis during PVR. Furthermore, utilizing collagen contraction assay, we demonstrate that TAK1 is a general regulator of the fibrotic response in RPE cells. On the whole, the study presented here establishes TAK1 as a novel player in the EMT process and reveals its crucial role in the homeostasis of RPE cells upon RD.

The ability to halt the process of EMT in RPE cells may reduce the severity of the fibrotic response that occurs upon PVR, lead to a better prognosis and increase the probability of successful surgery to reattach the retina.

## Materials and Methods

### Cell culture and treatments

Human RPE cell line (ARPE-19) (a gift from prof. Ruth Ashery-Padan[22]) was maintained in a 1:1 mixture of Dulbecco’s modified Eagle’s medium and Ham’s F12 Nutrient Mixture (DMEM/F12) containing 2 mM glutamine, 30 μg/mL penicillin, 50 μg/mL streptomycin and 10% fetal bovine serum (FBS) (all from Biological Industries, Kibbutz Beit Ha-Emek, Israel) and subcultured every 3 or 4 days in 100-mm dishes and grown in DMEM/F12 medium.

For the various treatments RPE cells were seeded in 60-mm, 6-well or 24-well plates (Corning, Amsterdam, Netherlands). Following attachment, the medium was replaced with serum-free medium for 16 hours. The cells were then treated for 1 hour with TAK1 inhibitor- 5Z-7-oxozeaenol (Sigma Aldrich, Rehovot, Israel) dissolved in Dimethyl sulfoxide (DMSO) according to the manufacturer's recommendation and diluted in serum-free medium to a final concentration of 1 μM, or treated with DMSO only (control). Alternatively, SB431542-TGF-β1 receptor kinase inhibitor (Sigma Aldrich, Rehovot, Israel) was used under the same conditions, at a final concentration of 10 μM. Finally the cells were exposed to TGF-β1 (PeproTech Asia, Rehovot, Israel) at a concentration of 2.5 ng/ml for the indicated times in each experiment.

### Immunofluorescence Staining

Human RPE cells (1×10^5^ cells/well) were seeded on fibronectin coated cover slips in DMEM/F12 and 10% FBS (in 6-well plates). Following starvation of 16 hours in serum free medium, the cells were treated with TGF-β1 (2.5ng/ml).

Cells were fixed with 4% paraformaldehyde for 20 min and permeabilized with 0.2% Triton-X100 for 5 min. Slides were stained with rabbit monoclonal phospho-TAK1 (Thr 187) (Bioss, Woburn, MA, USA), α-SMA (Sigma Aldrich, Rehovot, Israel), Isotype IgG2a, primary antibodies and then with Alexa Fluor 488-conjugated secondary antibodies (Millipore, Billerica MA, USA). F-actin was stained with TRITC-phalloidin and nuclei with DAPI (both from Sigma Aldrich, Rehovot, Israel). Images were de-convoluted and processed using a Zeiss laser confocal microscope (Zeiss, LSM-700, Oberkochen Germany).

### Protein Analysis

Cells were lysed in 50 mM Tris, 150 mM NaCl, 5 mM EGTA and 0.75% NP40 (pH 7.5), supplemented with Complete Protease Inhibitor Cocktail, 2 mM Na_3_VO_4_, 50 mM NaF, and 10 mM NaPPi. Cells debris was pelleted and protein concentration in the supernatant was determined using the BCA Protein Assay Reagent Kit (Pierce, Rockford, IL,USA). Proteins were separated by SDS−PAGE, transferred to a nitrocellulose membrane, and immunoblotted with the following primary antibodies: mouse monoclonal phospho-p38 (Thr180/Tyr182) antibodies, rabbit polyclonal p38 antibodies, rabbit monoclonal anti-phospho-Smad2 (Ser 465/467)/ Smad3(Ser 423/425), rabbit monoclonal anti Smad2/3, (all purchased from Cell Signaling, Danvers MA, USA), and monoclonal mouse GAPDH antibodies (Millipore, Billerica MA, USA). Following incubation with the appropriate secondary peroxidase-conjugated antibodies (Jackson, West Grove, PA, USA), immunopositive bands were visualized by an enhanced chemiluminescence (ECL) detection kit (Pierce, Rockford, IL, USA) using the Molecular Imager ChemiDoc XRS+ System (Bio-Rad, Hercules, CA, USA). The optical density of each protein band was quantified by the Image-Lab analysis software (Bio-Rad, Hercules, CA, USA). The amount of GAPDH in each lane was determined as a loading control.

### In Vitro Scratch Closure Assay

Human RPE cells (5×10^5^ cells/well) were plated on fibronectin coated 6-well plates, in DMEM/F12 regular medium. When the cells reached confluency the medium was replaced with serum free medium for 16 hours. Following starvation the cells were treated with 10 μg/ml mitomycin C (Sigma Aldrich, Rehovot, Israel) for 2 hours to prevent cell proliferation, and for one hour with TAK1 inhibitor (5Z-7-oxozeaenol, 1μM). Several scratches were then made in the cell monolayer, with a yellow pipette tip and growth medium was replaced with fresh serum free medium supplemented with or without TGF-β1 (2.5ng/ml). Cells migration towards gap closure was monitored by microscopy 0, 24, 48 and 72 h after wounding and photographed at low magnification (×200) under a light microscope (CKX41, Olympus, Airport City, Israel.). Gap width was measured and compared to day 0 using NIH imageJ software. (v1.47, NIH, Bethesda, MD, USA). All experiments were performed at least in triplicates.

### RNA extraction and cDNA synthesis

Total RNA was extracted from RPE cells using EZ-RNA kit (Biological Industries, Kibbutz Beit Ha-Emek, Israel). RNA was quantified by the NanoDrop ND-1000 UV-Vis Spectrophotometer at 260 nm (Thermo-scientific, Wilmington, DE, USA). 1 μg RNA was used for cDNA synthesis using the Thermo Verso qPCR kit (Thermo Scientific, Wilmington, DE, USA).

### Real Time Polymerase Chain Reaction (qPCR)

qPCR was performed using KAPA SYBR FAST qPCR Kit (Kapa Biosystems, Wilmington, MA,USA) and the Applied biosystem 7500 Real-Time PCR system. Assays for each sample and each primer set were performed in triplicate, using 1.5μl diluted cDNA and 0.3μM primers in a total reaction volume of 10 μl. The primers for CTGF: For 5’-GCAGGCTAGAGAAGCAGAGC-3’; Rev 5’-TGGAGATTTTGGGAGTACGG-3’. The primers for GAPDH: For 5'-TGGCCTCCAAGGAGTAAGAA-3’; Rev 5'-GGAAATTGTGAGGGAGATGC-3'. Cycling conditions were: activation 3 min at 95°C, followed by 35 cycles of denaturation for 3 sec at 95°C, annealing and extension 30 sec at 60°C and a melting curve program at 72–95°C. In the qPCR reaction the threshold was set at the start of the log-linear phase and kept constant for all the experiments using the same set of primers. The relative gene expression in different samples was calculated by extrapolation of the cycle threshold to a standard curve of the gene of interest and of GAPDH. Standard curves were performed in each experiment. All experiments were carried out in triplicates.

### Gelatin Zymography

Human RPE cells (1×10^5^ cells/well) were seeded on fibronectin coated 6-well plates in DMEM/F12 and 10% FBS. Following starvation of 16 hours in serum free medium, the cells were pre-incubated with 1 μM TAK1 inhibitor, 5Z-7-oxozeaenol or with 10mM TGF-β1 receptor inhibitor, SB431542 and treated with or without TGF-β1 (2.5ng/ml). Gelatin zymography was performed as described [23]. Briefly, culture media were collected after treatment and subjected to SDS-PAGE in 10% polyacrylamide gels copolymerized with 0.8 mg/ml gelatin. After electrophoresis, gels were incubated in 2.5% Triton X-100 (1 hour, 37°C) followed by overnight incubation at 37°C in 50 mM Tris-HCl (pH 7.8), 5 mM CaCl_2_, 0.02% NaN3, and 0.02% Brij. Gels were stained with 2.5% Coomassie Blue R-250 (Bio-Rad, Hercules, CA, USA) for 30 min followed by destaining in deionized water with 10% acetic acid and 20% methanol. Gels were scanned (miniBis pro, DNR, Jerusalem, Israel) and specific bands were quantified by the use of Quantity One 1-D analysis software (Bio-Rad, Hercules, CA, USA).

### Collagen contraction

Human RPE cells were resuspended at a concentration of 1.8x10^5^ cells/ml in DMEM X2 Medium and pre-incubated with 1 μM TAK1 inhibitor, 5Z-7-oxozeaenol, for 60 min or left untreated. Collagen (Biological Industries, Kibbutz Beit Ha-Emek, Israel) was then added to the cells at a concentration of 0.5 mg/ml, yielding a final cell concentration of 1.5x10^5^/ml in DMEM, 0.2% FBS and antibiotics. TGF-β was added to the cells to a concentration of 2.5 ng/ml and the suspension was seeded in a 24-well plate. After 1h incubation at 37°C for collagen polymerization, the gelatinous suspension was detached from the well edges by passing a sterile tip along the edge. Results were photo-documented at the indicated time points, and gel contraction was quantified using NIH imageJ software (v1.47, NIH, Bethesda, MD, USA).

### Quantification

Quantification of positive fluorescence cells was detected using a Meta laser-scanning confocal microscope. The pixel intensity of phoshpo-Thr 187- TAK1 and α-SMA was measured with ImageJ software, as pixel intensity (NIH, Bethesda, MD, USA).

### Statistics

Statistics were computed using Student’s *t*-test, two-tailed distribution. Values of P < 0.05 were considered significant.

## Results

### TGF-β1 Activates TAK1 MAP Kinase in RPE Cells

**Fig 1:**
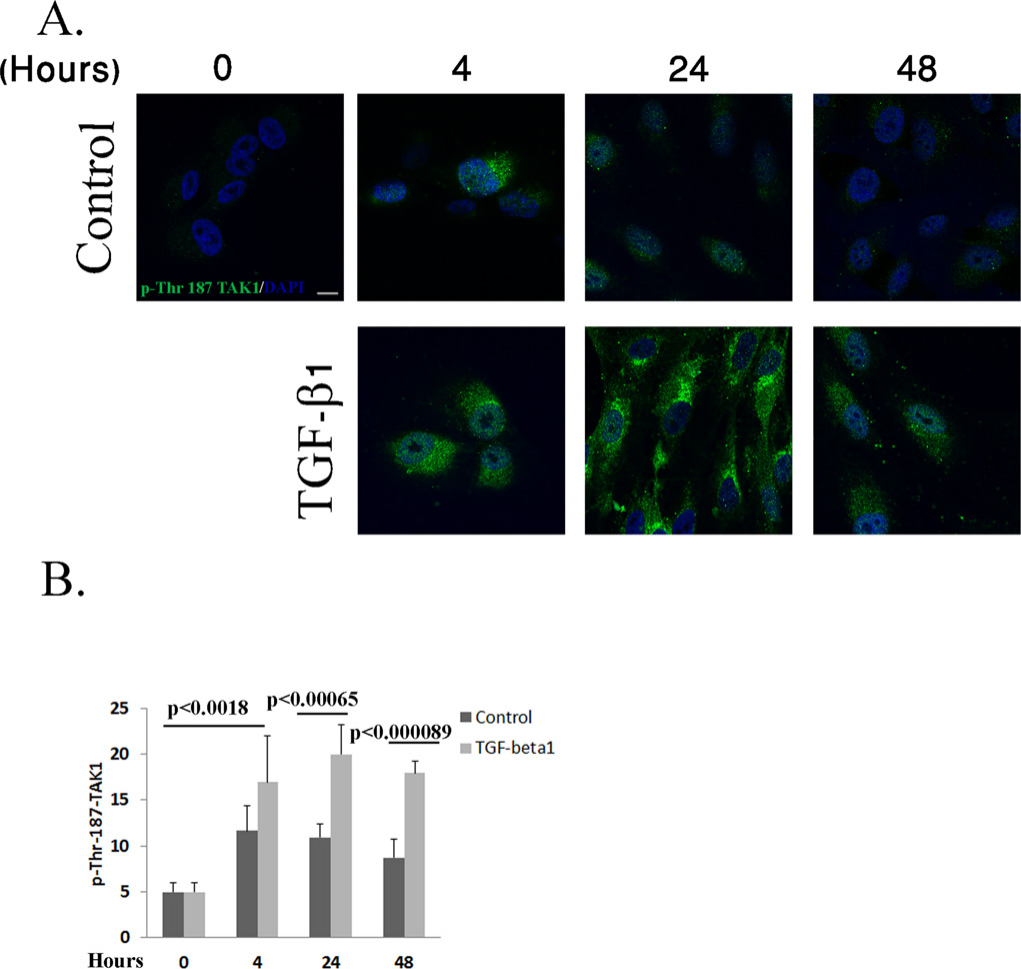
TAK1 is activated upon TGF-β1 stimulation in RPE cells. **A:** RPE cells were treated with TGF-β1 (2.5ng/ml) for the indicated times or left untreated. The cells were then immunostained with phospho-Thr 187 TAK1 antibodies (green) and DAPI (blue) as described in Materials and Methods. Representative photographs of three independent experiments. Scale bar is 10μm for all images. **B**: The histogram demonstrating pixel intensity, measured using Image-J software, is based on three independent experiments. (Number of cells: Control 0 = 24, Control 4 hours = 28, control 24 hours = 21, control 48 hours = 25; TGF-β1 4 hours = 26, TGF-β1 24 hours = 21, TGF-β1 48 hours = 22). Statistics were computed using student t-test (Two tailed distribution equal variance). Data is expressed as the Mean±SD.

To investigate the kinetics and the expression pattern of activated TAK1 in RPE cells upon TGF-β1 treatment, RPE cells were immunostained using phospho-TAK1 antibodies, which specifically recognize the activated form of TAK1 phosphorylated on threonine 187. As shown in Fig. 1, TAK1 activation gradually increased in the treated cells, reaching a peak 24 hours post treatment and then declined (Fig. 1A and 1B). In contract, the control cells displayed only marginal levels of activated TAK1, upon starvation, with no significant changes, thus demonstrating the specific activation of TAK1 by TGF-β1 in the RPE cells (Fig. 1A and 1B).

### Inhibition of TAK1 Halts the EMT Process upon TGF-β1 Stimulation in RPE cells

**Fig 2:**
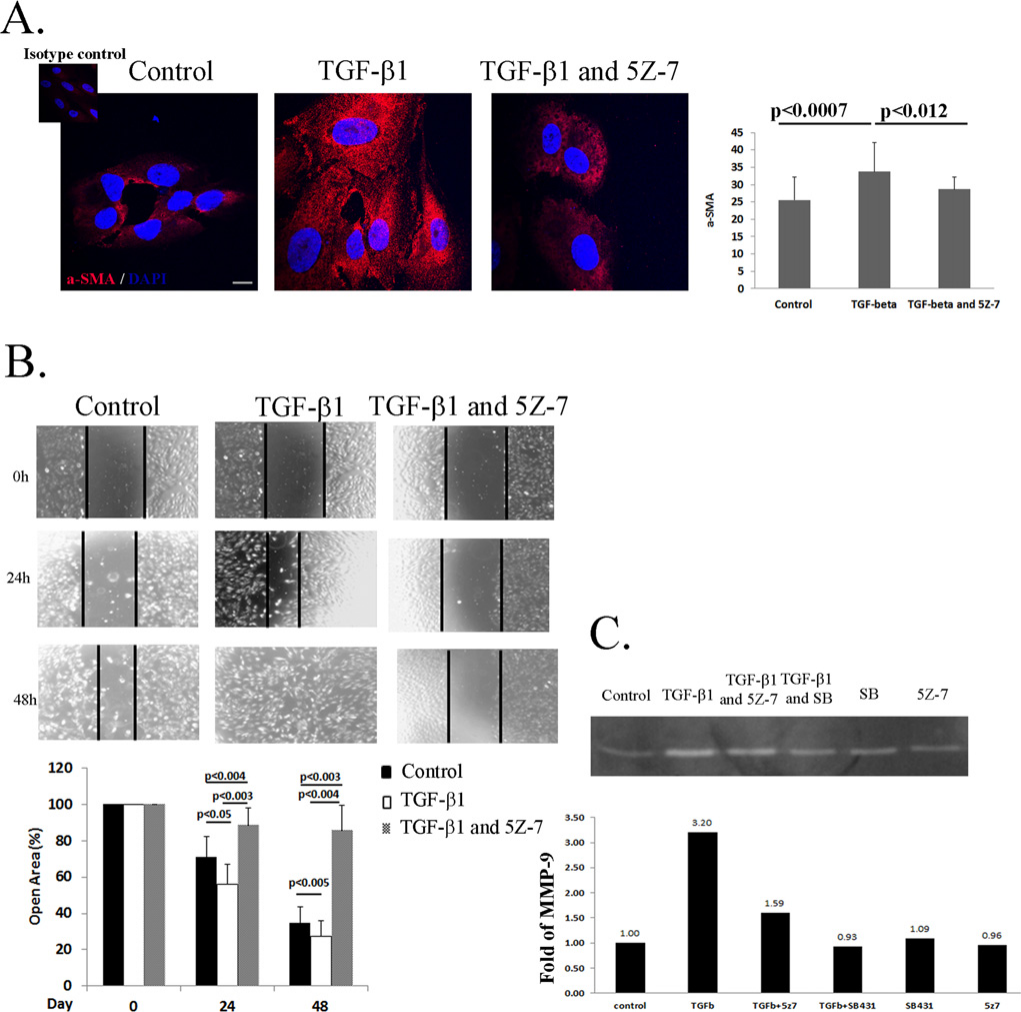
TAK1 regulates RPE cells EMT phenotype upon TGF-β stimulation. **A:** RPE cells were serum-starved for 16 hours, pretreated with 5Z-7-oxozeaenol (1μM) or left untreated for 1 hour. The cells were then treated with TGF-β1 (2.5ng/ml) as described in Materials and Methods for 24h and immunostained with α-SMA antibodies (red) and DAPI (blue). Representative photographs of three independent experiments. The histogram represent quantification of pixel intensity **B:** RPE cells were serum-starved for 16 hours, then treated with mitomycin C (10ng/ml) for 3h. Thereafter, pretreated with 5Z-7-oxozeaenol (1μM) or Dimethyl sulfoxide (DMSO) for 1 hour. Following this process a scratch was performed in the cell monolayer and the serum free medium was supplemented with TGF-β1 (2.5ng/ml) or left unsupplemented. Scratches were photo-documented at the indicated times (top panel) and their width was measured using Image-J software. The histogram (bottom panel) demonstrates the percentage of remaining gap at each time point, relative to initial gap width, based on three independent experiments. Bars are Mean±SD. **C:** RPE cells were serum-starved for 16 hours then pretreated with 5Z-7-oxozeaenol (1μM) or SB431542 (10μM), or DMSO for 1 hour. Finally, the medium was replaced with serum free medium with or without TGF-β1 (2.5ng/ml) and supernatants from the different treatments were collected after 24 hours. Total MMP-9 activities were processed by gelatin zymography (top panel) and the band intensity values were calculated by Quantity One 1-D analysis software. Histogram demonstrating secretion levels of MMP-9 in the different treatments (bottom panel). Statistics were computed using student t-test (Two tailed distribution equal variance). Data is expressed as the Mean±SD.

EMT of RPE cells is known to be mediated by TGF-β1 and characterized by increased expression α-SMA, enhanced cell migration and secretion of MMPs [4, 24]. To address a possible involvement of TAK1 in TGF-β1-induced RPE cells transdifferentiation, we studied the effects of specific TAK1 inhibitor on this process. A crucial step in transdifferentiation of RPE cells to myofibroblasts, is characterized by *de novo* synthesis of α-SMA [24]. To focus on this process *in vitro*, we stimulated RPE cells with TGF-β1 for 2 days under serum-free conditions with or without the presence of TAK1 inhibitor (5Z-7-oxozeaenol) or DMSO as a control (Fig. 2A). As can be seen in Fig. 2A, while TGF-β1 considerably stimulated α-SMA expression, the use of TAK1 inhibitor significantly attenuated this process (Fig. 2A).

Thus, demonstrating that aberrant activity of TAK1 might halt the EMT process of RPE cells.

To further support this finding we examined the migratory capacity of RPE cells, a well-known marker for EMT [25]. Cell migration was tested by the scratch assay, as described in Materials and Methods. The assay was performed on RPE cells pretreated or untreated with TAK1 inhibitor prior to stimulation with TGF-β1. As expected, addition of TGF-β1 significantly enhanced the migration of the RPE cells (Fig. 2B, TGF-β1) compared to control cells. At 24 hours, in the TGF-β1 treated culture, a major portion of the cells had already migrated into the wounded area, displaying almost complete wound closure at 48 hours. In contrast, the addition of 5Z-7-oxozeaenol to TGF-β1 stimulated cells abolished their migratory capacity.

Emerging evidence indicate that matrix metalloproteinases (MMPs) can stimulate processes associated with epithelial-mesenchymal transition (EMT) such as enhancement of cell invasion and migration[26]. Most reports suggest that predominance of MMP-2, -3 and -9 proteins correlate with worse prognosis[27]. Examining the role of TAK1 in the activation of MMP-9 by gelatin zymography demonstrated that after 24 hours of exposure, TGF-β1 stimulated 92-kDa MMP-9 protein activity in RPE cells (Fig. 2C). The activity of MMP-9 was significantly higher (3.2 fold) than the control group. Treatment of the cells with TAK1 inhibitor prior to TGF-β1 stimulation resulted in decreased MMP-9 activity, only 1.59 fold higher than the control group. Moreover, pre-treatment with SB431542, a specific inhibitor of the TGF-β1 receptor, completely abolished the activation of MMP-9. Thus, this result demonstrates that TGF-β1 is an inducer of MMP-9 activation in RPE cells and this phenomenon is mediated by TAK1.

**Fig 3:**
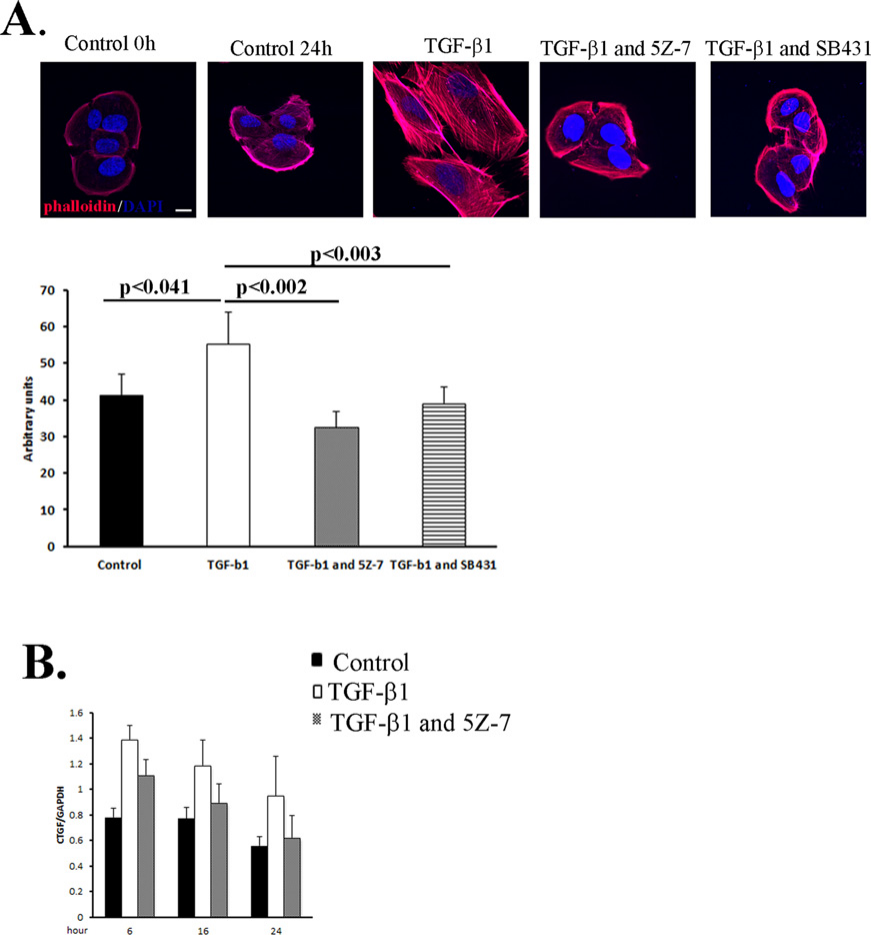
TGF-β regulates morphological and transcription changes in RPE cells through TAK1. **A:** RPE cells were plated on fibronectin-coated glass coverslips, serum-starved for 16 hours, pretreated with 5Z-7-oxozeaenol (1μM) or SB431542 (10μM), or DMSO for 1 hour, then treated with or without TGF-β1 for 2 days. Following treatment the cells were stained with rhodamine-phalloidin (actin fibers-red) and DAPI (blue) and visualized by confocal microscopy. Scale bars: 20μM. The histogram represents quantification of cells size **B:** Serum-starved RPE cells pretreated with 5Z-7-oxozeaenol (1μM) DMSO for 1 hour, were exposed to TGF-β as in A. Total RNA was extracted at each time point and qPCR was performed. Transcription levels of CTGF were determined after 6h, 16h and 24h. Bars represent the specific mRNA amount relative to GAPDH mRNA in the same samples. All experiments were performed in triplicates. Representative histogram from two independent experiments. Statistics were computed using student t-test (Two tailed distribution equal variance). Data is expressed as the Mean±SD.

In addition to the secretion of MMPs and increased migratory capacity, several morphological changes underlay EMT [8]. One of the phenotypes characterizing EMT is the increase in cells size mediated by TGF-β1 [8, 28]. Examination of RPE cells treated with TGF-β1 confirmed our expectations: cells were indeed enlarged 24 hours post treatment (Fig. 3A). In contrast, RPE cells that were treated with TAK1 inhibitor prior to TGF-β1 demonstrated minimal number of hypertrophy RPE cells. Concomitantly, we found that employing SB431542, a specific inhibitor of TGF-β1 receptor, abolished the increase in cell size similarly to the results with TAK1 inhibitor (Fig. 3A).

CTGF, an important stimulant of fibrosis, functions as a downstream mediator of TGF-β, stimulating cell proliferation and cell matrix deposition [29]. We examined the association between TGF-β1 and CTFG expression in RPE cells by Real Time PCR (Fig. 3B). Our results show that CTGF expression is significantly enhanced as early as 6 hours post TGF-β1 stimulation, and the effect lasts at least for 24 hours. Addition of the TAK1 inhibitor gradually reduced the levels of CTGF up to 24 hours post treatment, indicating that CTGF is a downstream mediator of TGF-β via TAK1 pathway (Fig. 3B).

### TAK1 Regulates TGF-β1 Signaling in RPE Cells

Our results indicated that inhibition of TAK1 reduced the expression of α-SMA and cell migration. These processes are known to be regulated in RPE cells by the TGF-β signaling through Smad2/3[14]. Therefore, this work examined whether TAK1 inhibition would have an effect on the phosphorylation of Smad2/3.

**Fig 4:**
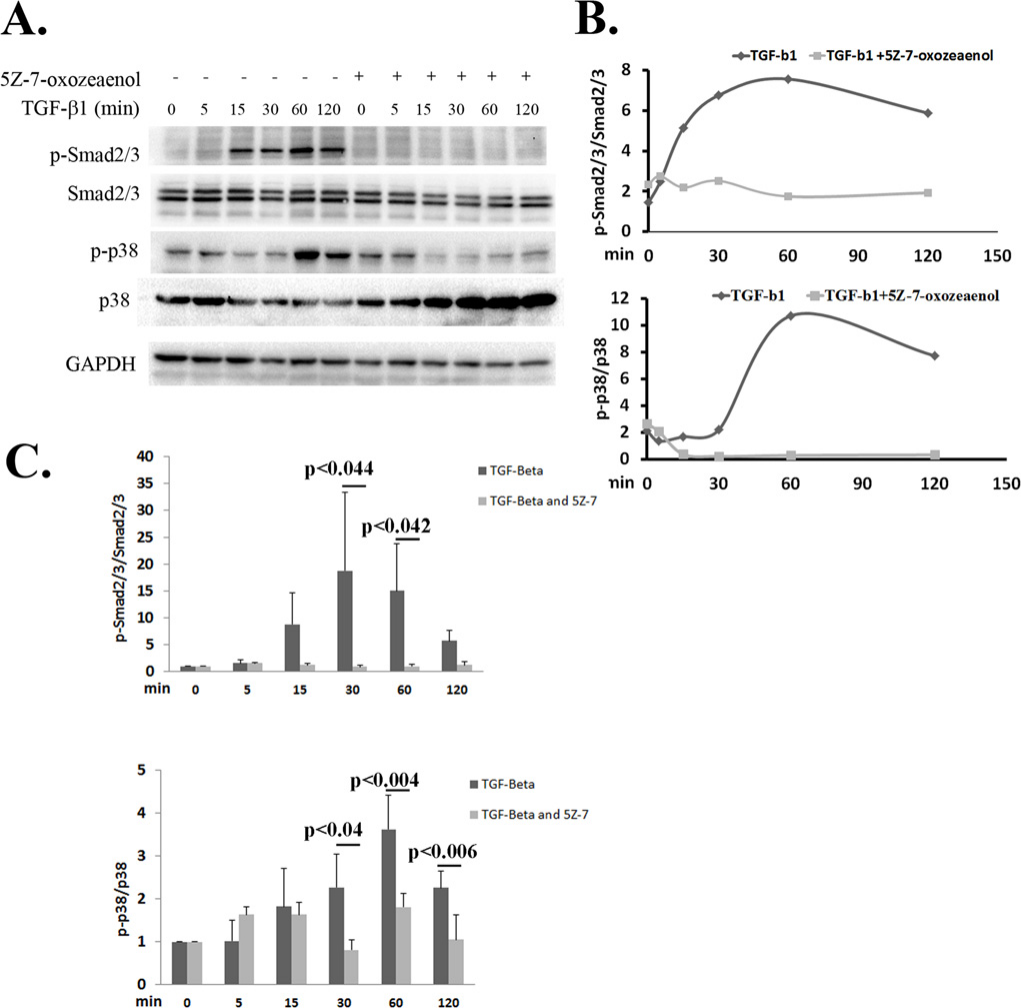
Inhibition of TAK1 abolishes the activation of TGF-β cascades. **A**: Serum-starved RPE cells were pretreated with or without with 5Z-7-oxozeaenol (1μM) for 1 hour and then with TGF-β (2.5ng/ml) for the indicated times. Total protein extracts were analyzed by western blot using the indicated antibodies. The blot shows a representative result of four independent experiments. **B:** Levels of phospho-Smad2/3 were quantified and normalized to total Smad2/3 **C:** Statistical analysis of p-p38 and p-Smad3 activation normalized to p38 and Smad3 respectively, with or without TGF-β stimulation. The histograms present results of 5 independent experiments. Statistics were computed using student t-test (Two tailed distribution equal variance). Data is expressed as the Mean±SD.

RPE cells were treated with or without 5Z-7-oxozeaenol followed by TGF-β treatment and Smad2/3 phosphorylation patterns were examined by immuno-blots. We observed that in TGF-β1 treated cells, phospho-Smad2/3 immediately increased and reached maximal activation at 60 minutes (Fig. 4A-4C). On the contrary, in cells that were pre-treated with 5Z-7-oxozeaenol Smad2/3 activation was barely detectable (Fig. 4A-4C). Calculating the ratio of phospho-Smad2/3 to total Smad2/3 at each time point clearly demonstrated that inhibition of TAK1 activity abolished Smad2/3 activation in RPE cells (Fig. 4B and 4C). Examination of the phosphorylation of p38, belonging to the non-canonical pathway of TGF-β1, revealed similar results to those obtained in the Smad2/3 signaling. Upon TGF-β1 stimulation, the phosphorylation of p38 increased, reaching a peak at 60 min (Fig. 4A-4C). In contrast, applying TAK1 inhibitor abolished p38 activation upon TGF-β1 stimulation (Fig. 4A-4C). These results demonstrate that inhibition of TAK1 halts both the canonical and non-canonical cascades of TGF-β.

### TAK1 Activity is Essential for EMT of the RPE Cells

**Fig 5:**
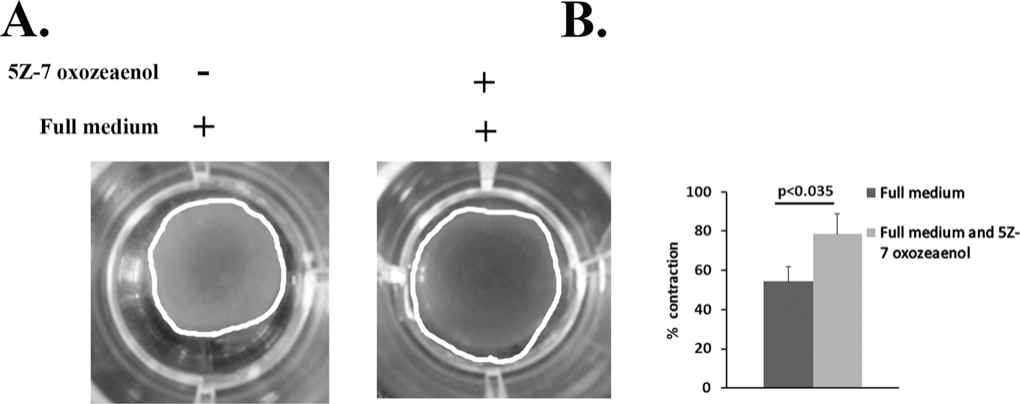
TAK1 is a general regulator of the EMT process in RPE cells. **A:** RPE cells were pre-treated with or without 5Z-7-oxozeaenol (1μM) and seeded in collagen lattices in full medium. The experiments were performed in triplicates. Lattices were photo-documented after 24 hours and measured using Image-J software. **B:** The histogram demonstrates the percentage of lattice area (marked by white line) relative to initial gel area, based on three independent experiments. Statistics were computed using student t-test (Two tailed distribution equal variance). Data is expressed as the Mean±SD.

The transition of RPE cells to myofibroblasts is characterized, among other aspects, by increased contractile activity [30–33]. To examine whether TAK1 is a general regulator of the EMT process affecting cell contractility, floating collagen matrix contraction assay was performed. The test was carried out in full serum with or without TAK1 inhibitor (Fig. 5). While in the control cells significant contraction was observed after 24 hours, reaching 50% of the initial size, TAK1 inhibition impaired the capacity of the cells to contract. The collagen lattice remained nearly 80% from the initial size (Fig. 5A and 5B).

Taken together our results demonstrate that the inhibition of TAK1 signaling prevented TGF-β1–induced RPE cells transdifferentiation with respect to α-SMA expression, increased cell contractility, cell migration and CTGF protein transcription.

## Discussion

Proliferative vitreoretinopathy (PVR) is a disease caused by the formation of fibrotic tissue on the detached retina, reducing its flexibility and in some cases preventing the reattachment of the retina [7, 34]. Although several other cell types, including Muller glia cells, are involved in the fibrotic reaction, RPE cells are the most critical contributor to the development of fibrous tissue on the retina [26]. In PVR, RPE cells are stimulated by inflammatory cytokines and chemokine factors, such as TGF-β1, to undergo EMT and to participate in epiretinal membrane formation [10]. Still, the precise mechanism of this process is not fully understood. In this work we reveal the involvement of TAK1 in the EMT process of RPE cells for the first time. The use of a specific inhibitor for TAK1, 5Z-7 oxozeaenol, abolishes all EMT characteristics found upon TGF-β1 stimulation. Furthermore, we demonstrate that TAK1 inhibition reduces the activation of Smad2/3 and p38, the canonical and non-canonical pathways of TGF-β1, respectively. Lastly, utilizing collagen contraction assay, this work shows that the role of TAK1 attenuating the fibrotic response of RPE cells is not restricted to the TGF-β1 pathway, but rather, TAK1 is a general inhibitor of this process.

### TAK1 Inhibition Prevents EMT of RPE cells

In addition to playing a crucial role in developmental processes, EMT is associated with fibrotic diseases and with cancer metastasis[5]. EMT and ectopic proliferation of RPE cells have been suggested to contribute to the development of PVR [4, 6, 28, 35]. When RPE cells become dislodged into the vitreous cavity or beneath the neurosensory retina, they experience an environmental change regarding exposure to cytokines and growth factors, and their normal cell-cell and cell-matrix interactions are disrupted. This process causes enhanced cell migration, high levels of α-SMA expression and increased contractility [36]. This work demonstrates that by inhibiting TAK1 activity these processes are significantly attenuated. Besides, specific inhibition of TAK1 maintains the quiescent and naive form of the RPE cells [6]. Furthermore, this work shows that the role of TAK1 in the process of EMT is not restricted to TGF-β1 signaling, rather it is a general function, as established by the collagen contraction assay. The event of impaired RPE contractility in the presence of TAK1 inhibitor occurs in full serum medium where all chemokines and cytokines play a part, thus demonstrating TAK1 general role in the process of EMT in RPE cells.

Our results are in accordance with a previous publication of Strippoli et al that demonstrated the critical role of TAK1 in EMT of peritoneal mesothelial cells (MCs) [37]. In the MCs TAK1 inhibition resulted in decreased migratory/invasive abilities of effluent-derived mesothelial cells. Moreover, they demonstrated that inhibition of TAK1 in mesenchymal-like mesothelial cells taken from the effluents of patients undergoing peritoneal dialysis, led to the acquisition of the apical to basolateral polarity, to increased expression of epithelial and to down-regulation of mesenchymal markers[37]. Earlier reports showed that TAK1 through the NF-kB signaling pathway triggered increased EMT and invasiveness in tumors[38]. In contrast, in human skin squamous cell carcinoma TAK1-deficient cells exhibited pronounced invasive morphology. These TAK1-deficient cancer cells adopt a more mesenchymal morphology characterized by a higher number of focal adhesions, increased surface expression of integrin α5β1 and active Rac1. Particularly, these mutant cells exert an increased response during TGFβ1-induced EMT [39]. TAK1 is upstream of relevant pathways such as TGF-β1 and is at the cross-road of different receptors, thus its involvement in EMT of numerous cells is critical for this process. In our system the role of TAK1 as an inducer of the EMT process is proven beyond doubt, illuminating the mechanism underlying EMT of the RPE cells.

### TAK1 Regulates the TGF-β1 Response in RPE Cells

TAK1 is known to act in the non-canonical pathway of the TGF-β response [14, 40]. By doing so, TAK1 controls the phosphorylation of downstream proteins such as p38 [41]. Indeed, we have shown that in RPE cells the activation of p38 is aberrant upon TAK1 inhibition. Following TGF-β1 stimulation p38 phosphorylation is enhanced up to 60 minutes after treatment. In contrast, employing 5Z-7-oxozeaenol (TAK1 specific inhibitor) diminishes this activity. Interestingly, a similar effect was found in the canonical cascades of the TGF-β1 response. While TGF-β1 treatment increased the activation of Smad2/3 up to 120 minutes, the inhibition of TAK1 activity abrogated Smad2/3 phosphorylation in TGF-β1 treated cells. These novel data emphasize the importance of TAK1 in the TGF-β1 response. In our system of RPE cells, TAK1 regulates not only the activation of the non-canonical cascade of TGF-β1 (p38), but also the canonical cascade, the Smad2/3 activation. Thus, the outcome of the TGF-β1 response in the RPE cells is TAK1 dependent. In mesothelial cells (MCs), TAK1 inhibition led to a reduction of both c-jun and Smad3 phosphorylation after treatment of TGF-β1 with IL-1β [37]. This phenomenon was also seen in neural crest cells [42]. Yumoto et al have found that TAK1 is required for appropriate activation of both p38 MAPK (non-canonical pathway) and R-Smads (canonical pathway) in the neural crest cells. To date, it is widely accepted that TAK1 controls p38 activation in the TGF-β response[43], whereas the question of TAK1 regulating the Smads activity is still object for debate. In the RPE cells, TAK1 inhibition led to a decrease of both p38 and Smad2/3 phosphorylation. This work demonstrated that TGF-β1 stimulation caused the activation of both proteins. Conversely, employing 5Z-7-oxozeaenol abolished the phosphorylation of both cascades, resulting in arrest of the EMT process. It is well known that the use of 5Z-7-oxozeaenol directly prevents the activation of p38 by TAK1 [44]. However, the effect of TAK1 on the activity of Smad2/3 is probably indirect. Several studies have attempted to elucidate the effect of the non-canonical cascade on the canonical cascade of TGF-β [45]. Early reports demonstrated that p38 could influence the activity and phosphorylation of Smad2/3 [46]. Moreover it was suggested that TAK1 may also affect Smad3 activity indirectly, inducing the degradation of the Smad3 inhibitor SnoN [16]. Collectively, our data put emphasis for the first time on the importance of TAK1 in regulating TGF-β1 signaling pathways in RPE cells. TAK1 emerges as an essential player in the TGF-β response, regulating the canonical and non-canonical cascades. It should be noted that this work has been performed utilizing ARPE-19, a well-known and acceptable model system for RPE cells. However, in the literature the physiology of these cells is still being studied. Several studies have shown that the ARPE-19 cells are polarized [47–49] and in contrast other showed they are not [50], thus raising the possibility that these cells are not mimicking the *in vivo* RPE cells. Nevertheless, this issue in not in the scope of this work and needs to be further studied.

Considering all the above together with other findings lead us to hypothesize that inhibition of TAK1 MAP Kinase may be beneficial in preventing and treating PVR. Although PVR is caused by the activation of many cell types, such as retinal glial components and other cells, activation and fibroblastic transformation of RPE cells is the critical feature in the development of this disease.

Current therapies aimed to limit or reverse PVR have failed to achieve significant clinical benefits, highlighting the need for further research to elucidate the basic mechanisms underlying PVR. Our data that demonstrate the critical role of TAK1 in transformation of RPE cells can be used as a novel therapeutic avenue for PVR and subsequently to prevent blindness.
